# Unexpected Exacerbation of Neuroinflammatory Response After a Combined Therapy in Old Parkinsonian Mice

**DOI:** 10.3389/fncel.2018.00451

**Published:** 2018-11-30

**Authors:** Ana Luisa Gil-Martínez, Lorena Cuenca, Cristina Estrada, Consuelo Sánchez-Rodrigo, Emiliano Fernández-Villalba, María Trinidad Herrero

**Affiliations:** ^1^Clinical and Experimental Neuroscience Group (NiCE-IMIB), Department of Human Anatomy and Psychobiology, Institute for Aging Research, School of Medicine, University of Murcia, Murcia, Spain; ^2^Biomedical Research Institute of Murcia (IMIB-Arrixaca), Campus of Health Sciences, University of Murcia, Murcia, Spain

**Keywords:** aging, drug-repositioning, glia, neuroinflammation, oxidative stress, Parkinsonism

## Abstract

The design of therapeutic strategies that focus on the repositioning of anti-inflammatory and antioxidant drugs are a great bet to slow down the progression of neurodegenerative disorders. Despite the fact that Parkinson’s disease (PD) is an age-related pathology, almost all experimental studies are carried out in young animals. Here, we evaluated the possible neuroprotective effect of the combination of the antioxidant N-acetylcysteine (NAC) and the anti-inflammatory HA-1077 in aged 1-methyl-4-phenyl-1,2,3,6-tetrahydropyridine (MPTP)-treated mice (C57BL/6 mice, 20 months old), whose individual treatment has been shown to have neuroprotective effects in this Parkinsonism model. Interestingly, NAC+HA-1077-based treatment produced a significant increase in dopaminergic neuronal death accompanied by an increase in microglial and astroglial activation in the Substantia Nigra *pars compacta* (SNpc) and striatum of old-Parkinsonian mice compared to their control group. The astroglial response was also explored by co-immunostaining for GFAP and S100b together with p-JNK and it was found to be particularly exacerbated in the MPTP+NAC+HA-1077 group. The unexpected toxic effects found in the combined use of NAC and HA-1077 in old-Parkinsonian mice highlight the importance of taking into account that in elderly Parkinsonian patients the combination of some drugs (most of them used for other different age-related alterations) can have side effects that may result in the exacerbation of the neurodegenerative process.

## Introduction

Parkinson’s disease (PD) is a pathology based on the chronic and the progressive loss of dopaminergic neurons in the Substantia Nigra *pars compacta* (SNpc) that results in a decrease of dopamine levels in the nigrostriatal pathway. This fact implies the development of motor alterations as stiffness, bradykinesia, resting tremor and postural instability. PD is the second most prevalent neurodegenerative disorder that affects 100–200 per 100,000 people at the age of 65–70 years (Tysnes and Storstein, 2017). Although the cause of the disease is not clear yet, many studies emphasize aging as the main risk factor that contributes to the development of the disease (Collier et al., 2017) over other described risk factors, as genetic causes (Kalia and Lang, 2015) or exposure to environmental neurotoxins such as 1-methyl-4-phenyl-1,2,3,6-tetrahydropyridine (MPTP) (Kopin and Markey, 1988).

In this line, it is crucial to take into account the close relationship between aging and PD to understand the progression of the disease. An elderly condition exacerbates the main pathogenic pathways described as etiological bases of PD such as mitochondrial dysfunction (Bose and Beal, 2016), dysregulation of protein homeostasis (Sin and Nollen, 2015) and oxidative stress (Gaki and Papavassiliou, 2014). However, aging is a variable hardly ever incorporated in the studies with experimental animals as it entails a risk in the subjects’ mortality and an increase in the associated costs. Only a few *post-mortem* analysis have been carried out in PD patients with genetic predisposition along with age-associated immunological alterations that have demonstrated an association between slight inflammation in the CNS and vulnerability to the degeneration of dopaminergic neurons (Tiwari and Pal, 2017). In the CNS, glial cells are the main responsible in the immune response. Firstly, after an injury microglial cells are activated and they migrate to the damaged area to phagocytize the apoptotic cells. If the pathological condition progresses, there is an imbalance of the pro-inflammatory over the anti-inflammatory processes that triggers the exacerbation of the response, probably activating astrocytes (Halliday and Stevens, 2011). Both PD and aged brains seem to share a similar inflammatory condition entitled “neuro-inflammaging,” characterized by complex processes where astrocytes and microglial cells chronically produce several toxic agents (i.e., pro-inflammatory cytokines) which damage neighboring neurons (Rodriguez et al., 2015).

Different protein families mediate the activation and maintenance of glial-related processes (Dagda et al., 2009). Among them, we highlight the signaling pathway of the mitogen-activated protein kinases (MAPKs). This family is composed of an extracellular signal-regulated kinase (ERK), c-Jun NH2-terminal kinase (JNK), and p38 MAPK. In the last years, MAPKs have been shown to be involved in the development of some neurodegenerative diseases such as Alzheimer’s or Parkinson’s disease. The role of JNK is interesting as it has been demonstrated to be involved in the activation of cellular processes such as the release of pro-inflammatory cytokines and the oxidative stress response mediated by microglia and astroglia, thus contributing to the progression of neuroinflammation (Kim and Choi, 2015).

Considering the important role of inflammatory processes and oxidative stress, different research groups around the world focus their efforts on the study of the effect of anti-inflammatory drugs on dopaminergic neuronal death. In recent years, a new therapeutic strategy called ”drug repositioning” has been introduced for the design and development of new treatments for PD patients. Drug repositioning consists of refocusing medicines indicated for other pathologies that have been demonstrated to have a positive effect in PD development (Brundin et al., 2015). The main advantage of this strategy is the availability of human clinical studies about the reliability and safety of these drugs (Maiti et al., 2017). This issue becomes especially controversial in PD and other age-related disorders whose patients are poly-medicated, both for the treatment of the disease and for the age-associated problems. The elderly patients have a less plastic brain and therefore, are more vulnerable to the severe toxicity produced by drug interaction (Savva et al., 2009).

Based on the previously described data, in the present study, we wanted to analyze the effect of an anti-inflammatory (fasudil, HA-1077) and antioxidant (N-acetylcysteine, NAC) in old-Parkinsonian mice. Both drugs have been shown to have an individual beneficial effect on both dopaminergic neuronal death and glial response. HA-1077 is a ROC-kinase inhibitor, a protein that is essential for the motility and phagocytic capacity of microglia. Previous studies from our research group showed that the use of HA-1077 decreases the activation of microglia in MPTP-intoxicated mice (Barcia et al., 2012). Additionally, Pan and co-workers demonstrated that treatment with the clinical commonly used antioxidant N-acetylcysteine (NAC), an inhibitor of the activation of JNK signaling pathway, has a protective effect on dopaminergic neuronal death in the young MPTP mouse model (Pan et al., 2009). Thus, our aim was to study the synergistic effect of a combined treatment of NAC and HA-1077 on dopaminergic neuronal death and inflammatory processes in old mice intoxicated with MPTP.

## Materials and Methods

### Animals

The studies were performed on 64 male elderly C57BL/6J mice (20 months of age, weight 27–29 g) provided by Eleverge Janvier (Le Genest St Isle, France) and maintained in an isolated room with access to water and food *ad libitum*, under ambient conditions (20 ± 2°C) and light cycles: darkness of 12:12 h. All animal testing methods and procedures were approved by the Council of the European Community Committee (2010/63/EU) and by the Institutional Committee on Animal Ethics of the University of Murcia (REGA ES300305440012).

### Experimental Design and Animal Model of Parkinsonism

**FIGURE 1:**
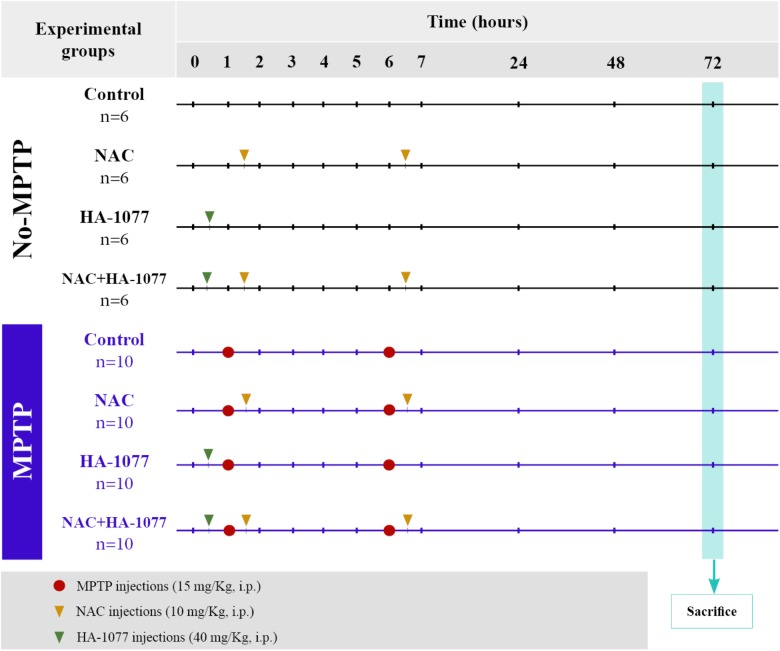
Scheme of the experimental design and distribution of the groups and animals with their respective treatments.

Animals were divided into two main groups: non-MPTP group (*n* = 24) and MPTP group (*n* = 40). Parkinsonism was induced via intraperitoneal (i.p.) injection of the neurotoxin 1-methyl-4-phenyl-1,2,3,6-tetrahydropyridine (MPTP, Sigma-Aldrich), which specifically causes dopaminergic neuronal death. The dose administered per animal was 30 mg/kg, divided into two injections of 15 mg/kg each and spaced 5 h over a day (Figure 1; Jackson-Lewis and Przedborski, 2007; Annese et al., 2015).

### Drug Administration

The animals belonging to the two main groups (both non-MPTP and MPTP) were divided into four subgroups according to the treatment: (i) control (untreated); (ii) NAC; (iii) HA-1077; (iv) NAC+HA-1077. NAC treated groups received a dose of 100 mg/Kg (i.p.) of NAC (Sigma Aldrich) half an hour after each injection of MPTP (NAC and MPTP+NAC and MPTP+NAC+HA-1077), following the protocol described by Pan et al. (2009). A dose of 40 mg/Kg (i.p.) of HA-1077 (Sigma-Aldrich) half an hour before the first MPTP injection were administrated to HA-1077, MPTP+HA-1077, and MPTP+NAC+HA-1077 groups (Figure 1), following the protocol described by Barcia et al. (2012).

### Sample Preparation

Seventy two hours after the drug treatment, the animals were sacrificed by cervical dislocation under an overdose of ketamine (50 mg/Kg, Imagene, Merial) and Xylazine (50 mg/Kg, Xilagesic, Calier Laboratories). For immunohistochemistry and immunofluorescence, brains were extracted, fixed overnight at 4°C in 4% paraformaldehyde in phosphate buffered saline or PBS (0.1 M, pH 7.4), washed with ethanol and embedded in paraffin. For Western Blot and ELISA, brains were extracted and immediately frozen. The cerebellum, midbrain and striatum were dissected according to the coordinates of the mouse brain atlas (Franklin and Paxinos, 2008). The tissue was homogenized with RIPA lysis buffer: 50 mM Tris-HCl pH 7.6, 150 mM NaCl, 5 mM EDTA, 1% Triton X-100, 1% SDS, 50 mM NaF, protease and phosphatases inhibitors (Abcam). Tissue extracts were incubated 2 h at 4°C under constant stirring. Samples were centrifuged (13,000 rpm, 20 min at 4°C) and the supernatant was collected. Total protein concentration was determined (Pierce BCA Protein Assay Kit, Thermo Scientific) and Western Blot and ELISA were performed according to the protocol detailed above.

The number of animals used for immunohistochemical (IHQ)/immunofluorescence (IF) analysis was *n* = 3 in the No-MPTP groups (control, NAC, HA-1077, NAC+HA-1077) and *n* = 5 in the MPTP groups (control, NAC, HA-1077, NAC+HA-1077). For Western Blot and ELISA experiment, the number of animals used was *n* = 3 in the No-MPTP groups (control, NAC, HA-1077, NAC+HA-1077) and *n* = 5 in the MPTP groups (control, NAC, HA-1077, NAC+HA-1077).

### Immunohistochemistry and Immunofluorescence

Coronal sections (7 μm) of SNpc and striatum were cut on microtome (Thermo Scientific HM 325 Rotary Microtome, Thermo Fisher Scientific). The sections were deparaffinized in xylene, rehydrated in a gradient of ethanol (100, 95, and 80%) and distilled water. Antigenic retrieval was performed in citrate buffer 30 min at 95°C (10 mM citric acid, pH 6.0). In immunohistochemistry, endogenous peroxidase was inhibited with a solution of 0.3% H_2_O_2_ for 20 min and non-specific binding of the antibodies was blocked (both in immunohistochemistry and immunofluorescence) in a blocking solution for 30 min: tris-buffered saline 0.1 M pH 8.4 (TBS_IHQ_), 10% goat serum and 0.5% Triton X-100. After two washes with TBS_IHQ_ the samples were incubated with the corresponding primary antibody overnight at 4°C (Table 1). Primary antibodies used in immunohistochemistry and immunofluorescence were diluted in TBS_IHQ_+Tween 0.5%+1% goat serum at the dilution indicated in Table 1. Following the primary antibody, two washes were performed with TBS_IHQ_ and sections were incubated with the secondary antibody diluted in TBS_IHQ_ at the concentrations indicated in Table 1 for 30 min in immunohistochemistry (4 h in immunofluorescence). Sections were washed with TBS_IHQ_ and incubated at room temperature (RT) with avidin/biotin conjugated to peroxidase (ABC Elite Kit, Vector Laboratories), staining using the kit 3,3′-diaminobenzidine (DAB Peroxidase HRP Substrate Kit, Vector Laboratories) and, finally, assembly was performed for observation under the optical microscope. In the case of immunofluorescence, after washing with TBS_IHQ_ after incubation with the secondary antibody, the samples were assayed (VECTASHIELD Antifade Mounting Medium, Vector Laboratories).

**Table 1:** Antibodies and protocols for the different techniques.

Primary antibodies	Host, code	Application	Antibody supplier, dilution, incubation period	Secondary antibodies, code	Antibody supplier, dilution, incubation period
Anti-TH	Mouse MAB318	IHQ^a^	Millipore, 1:500, ov	Anti-IgG mouse (Biotynilated) BA-9200	Vector Laboratories, 1:250, 1 h
		WB^c^	Millipore, 1:5000, ov	Anti-IgG mouse (HRP) 115-035-003	Jackson Immunoresearch, 1:5000, 2 h
		IF^b^	Millipore, 1:500, ov	Double Labeling Kit^d^ DK-8818	Vector Laboratories, 30 min
Anti-Iba1	Rabbit B178846	IHQ^a^	Abcam, 1:1000, ov	Anti-IgG rabbit (Biotynilated) BA-1000	Vector Laboratories, 1:250, 1 h
		IF^b^	Abcam, 1:1000, ov	Double Labeling Kit^d^ DK-8818	Vector Laboratories, 30 min
Anti-GFAP	Mouse MAB360	IHQ^a^	Millipore, 1:500, ov	Anti-IgG mouse (Biotynilated) BA-9200	Vector Laboratories, 1:250, 1 h
		IF^b^	Millipore, 1:500, ov	Double Labeling Kit^d^ DK-8818	Vector Laboratories, 30 min
	Rabbit AB7260	IF^b^	Millipore, 1:500, ov	Double Labeling Kit^d^ DK-8818	Vector Laboratories, 30 min
Anti-S100b	Rabbit AB52642	IF^b^	Abcam, 1:500, ov	Double Labeling Kit^d^ DK-8818	Vector Laboratories, 30 min
Anti-JNK3	Rabbit AB87404	WB^c^	Abcam, 1:5000, ov	Peroxidase anti-rabbit IgG 111-035-144	Jackson Immunorearch, 1:5000, 2 h
Anti-GADPH	Mouse AB9684	WB^c^	Abcam, 1:5000, ov	Anti-IgG mouse (HRP) 115-035-003	Jackson Immunorearch, 1:5000, 2 h
Anti-Phopho-SAPK/JNK	Mouse #9255	IF^b^	Cell signaling, 1:200, ov	Double Labeling Kit^d^ DK-8818	Vector Laboratories, 30 min


*^a^Immunohistochemistry. ^*b*^Immunofluorescence. ^*c*^Western Blot. ^*d*^VectaFluor Duet Immunofluorescence Double Labeling Kit, DyLight 488 Anti-Rabbit (green)/DyLight 594 Anti-Mouse (red).*

### Quantification of DAB and Immunofluorescence Labeling

For DAB labeling quantification, images were obtained by Hall 100 ZEISS optical microscope with an Axiocam ZEISS digital camera and were analyzed using the NIH ImageJ software (ImageJ; NIH, Bethesda, MD, United States). Field size was set at 1088 × 1040 pixels. All the analysis were performed without post-processing the images.

SNpc and striatum areas were delimitated according to anatomical coordinates (Franklin and Paxinos, 2008). For each immunolabeling, eight serial sections of the SNpc and striatum of each animal were used at different rostrocaudal levels (1 section in every 10) and quantified following the conditions reported by Blesa et al. (2012). The serial sections used for the SNpc analysis were located in the anatomical coordinates between bregma -2.06 and -3.80 mm and for the striatum from bregma 0.02–0.26 mm (for more details see Supplementary Figures S1, S2). The quantification of all immunohistochemical analysis were performed in the striatum (both hemispheres in each slice) in micrographs covering the whole surface area of the dorso-lateral region taken with the 20× magnification and using the principle of the optical dissector (Sterio, 1984). Positive cells were counted only when they touched the superior and left limit of the square. A representative image of the No-MPTP groups has been shown for each marker in the Supplementary Figure S3.

#### Quantification of TH-Positive (TH+) Cells

In SNpc, the total number of TH+ cells was determined using the 20× objective and results are expressed as the number of TH+ cells/mm^3^. Counting unit was the nucleus surrounded by immunoreactive cytoplasm. The area of the SNpc was calculated by a systematic non-biased method. In the striatum, the dopaminergic innervations were quantified by measuring the optical density (O.D.) of DAB signal, expressed as Area (%)/mean gray value (Barcia et al., 2005).

#### Quantification and Stereological Analysis of Microglia

The study of microglia cells was performed using the marker Iba-1. Iba-1+ cells were counted in the SNpc and in the striatum and were expressed as Iba-1+ cells/mm^3^. Counting unit was the nucleus surrounded by immunoreactive cytoplasm.

#### Quantification of GFAP+ Cells (Astrocytes) and Stereological Analysis of GFAP+ Astroglia

The number of GFAP+ cells in SNpc and striatum was determined following the same procedure as the one described for microglia analysis but using as counting unit the nucleus surrounded by GFAP-immunoreactive cytoplasm, and expressed as GFAP+ cells/mm^3^.

#### Iba-1+ and GFAP+ Cells Morphological Analysis

For microglia and astroglia morphological analysis at SNpc and striatum level, immunolabeling of Iba-1 and GFAP was performed. For each label, we acquired z-stack images at 0.5 μm intervals using a confocal microscope Leica TCS-SP8 (SACE, University of Murcia) with the 63× glycol-immersion objective lens at optical zoom of 0.75 (x-axis x y-axis, 1024 × 1024 pixels). In order to avoid quantification and measurement bias, all the images were obtained under the same setting conditions. Morphological analysis was performed by O.D. measure following the protocol described by Otsu (1979) and, subsequently, developed by Tatsumi et al. (2016). We used ImageJ software (NIH, Bethesda, MD, United States) to convert the images to 8 bit grayscale images and to determine mean O.D. values. For each immunolabeling and area (GFAP or Iba-1, SNpc or striatum), 1/4 images out of a total 24 images in three different regions of each animal were measured to obtain the average O.D. value of the z-stack images.

### Western Blot

Equal amounts of protein (30 μg) from each tissue extract were resolved in 12% polyacrylamide gels under denaturing conditions at constant voltage (110 V) at RT. After electrophoresis, proteins were transferred to PVDF membranes (Immobilon, Millipore) at constant voltage (20 V, 50 min, Trans-Blot SD Semi-Dry Transfer Cell, Bio-Rad) using the transfer buffer: 25 mM Tris-HCl, 192 mM glycine, 0.1% SDS, 20% methanol. Membranes were incubated for 1 h and constant stirring in blocking solution: 0.1 M TBS pH 7.5 (TBS_WB_) + 0.05% Tween-20 (TBST) + 5% BSA. Membranes were then incubated with the specific primary antibody at 4°C overnight and constant stirring (Table 1). Primary antibodies used in Western Blot were diluted in TBST + 3% BSA at the dilution as referred in Table 1. Primary anti-glyceraldehyde-3-phosphate dehydrogenase (GADPH) antibody was added as the internal loading control of protein. After incubation with the primary antibody and 3 washes with TBST, the membranes were incubated with the corresponding peroxidase-conjugated secondary antibody (Table 1) diluted in TBST, at RT and constant agitation for 2 h. Protein detection was performed using the 3,3′-diaminobenzidine kit (DAB Peroxidase HRP Substrate Kit, Vector Laboratories). For TH, a specific band of approximately 60 kDa was detected in the SNpc and three specific bands of 55–60 kDa were detected at striatum level. The signal quantification was performed in the 60 kDa band as it has been described to be the functional isoform of the protein (Tabrez et al., 2012).

To calculate the intensity of the bands the membranes were scanned and analyzed by densitometry analysis using the ImageJ software. The optical density of each band corresponding to the protein of interest was normalized individually with the optical density of the GADPH band obtained for the sample from the same well. Densitometric analysis and quantification of the protein bands was performed using the ImageJ software.

### ELISA

Total JNK1/2 levels and phosphorylated-JNK1/2 levels were measured using Enzyme-Linked Immunoabsorbent Assay (ELISA). The assay was conducted using JNK1/2 (pT183/Y185) + Total JNK1/2 SimpleStep ELISA^TM^ Kit (Abcam) according to the manufacturer’s protocols. Signal generated was read as absorbance at 450 nm using CLARIOstar reader (BMG LABTECH).

### Data and Statistical Analysis

Data are shown as mean ± SD. The statistical analysis were performed with GraphPrism 7.0 software using a two-way ANOVA test followed by *post hoc* analysis Sidak. The null hypothesis was rejected considering values of *p* < 0.05 in all cases.

## Results

### The Combined Treatment NAC+HA-1077 Does Not Prevent Dopaminergic Neuronal Death in Parkinsonian Mice

**FIGURE 2:**
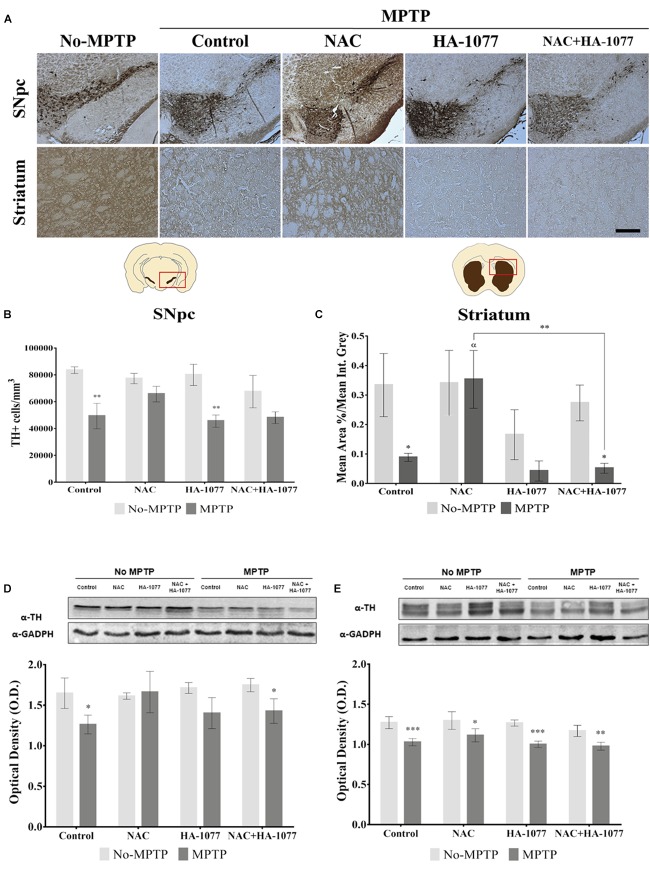
Effect of the different treatments (NAC, HA-1077 and NAC+HA-1077) on dopaminergic neuronal death. **(A)** Representative micrographs of TH immunostaining in coronal sections at the SNpc and striatum levels: No-MPTP, MPTP, MPTP+NAC, MPTP+HA-1077, MPTP+NAC+HA-1077 (Magnification 10×, Scale bar = 100 μm). **(B,D)** Quantification of TH expression in SNpc by immunohistochemical analysis. **(B)** Very significant increase in dopaminergic neuronal death was in MPTP (^∗∗^*p* = 0.0026) and MPTP+HA-1077 (^∗∗^*p* = 0.0025) compared with their No-MPTP groups. **(D)** Western Blot results showed a significant decrease in TH expression in MPTP (^∗^*p* = 0.0101) and MPTP+NAC+HA-1077 (^∗^*p* = 0.0221). **(C,E)** Quantification of TH expression in the striatum. **(C)** Immunohistochemical analysis showed a significant decrease of TH expression in the innervations of the dopaminergic neurons in MPTP (^∗^*p* = 0.0126) and MPTP+NAC+HA-1077 groups (^∗^*p* = 0.0117) compared with their control groups. MPTP+NAC vs. MPTP (α = 0.0286) and MPTP+NAC vs. MPTP+NAC+HA-1077 (^∗∗^*p* = 0.0046). **(E)** Quantification by Western Blot of TH protein expression showed significant differences in MPTP+NAC (^∗^*p* = 0.0103) and a very significant in MPTP (^∗∗∗^*p* = 0.0003), MPTP+HA-1077 (^∗∗∗^*p* = 0.0006) and MPTP+NAC+HA-1077 (^∗∗^*p* = 0.0076) compared with their No-MPTP groups.

To verify the possible neuroprotective effect of the combined treatment NAC+HA-1077 on the loss of dopaminergic neurons, immunohistochemical staining of TH in SNpc and in the striatum was performed (Figure 2A). As shown in Figure 2, dopaminergic neuronal death at the SNpc level was statistically very significant in the MPTP group (*p* = 0.0026) compared to the control group. These results validated our model for Parkinsonism. Similar results were obtained for the dopaminergic cell loss of the MPTP+HA-1077 group compared to its control (*p* = 0.0025). Surprisingly, no significant differences were observed in the TH+cells/mm^3^ in the MPTP+NAC+HA-1077 group compared to the control group (Figure 2B). The levels of TH expression in the striatum were significantly decreased in the MPTP (*p* = 0.0126) and MPTP+NAC+HA-1077 (*p* = 0.0117) groups compared to their respective No-MPTP groups (Figure 2C). In contrast, TH levels in the striatum of the MPTP+NAC group were similar to those in the control group and significantly higher than in the MPTP group (*p* = 0.0286). These results demonstrate for the first time that NAC has a neuroprotective effect on dopaminergic neuronal death in old mice after MPTP insult, opening the action spectrum described by Pan et al. (2009). Western Blot analysis for TH expression (Figures 2D,E) reinforced the results obtained by immunohistochemical assay in which MPTP and MPTP+NAC+HA-1077 groups showed significantly decreased levels compared to their controls.

### Treatment With NAC+HA-1077 Exacerbates Microglial Response in the MPTP Parkinsonism Model

**FIGURE 3:**
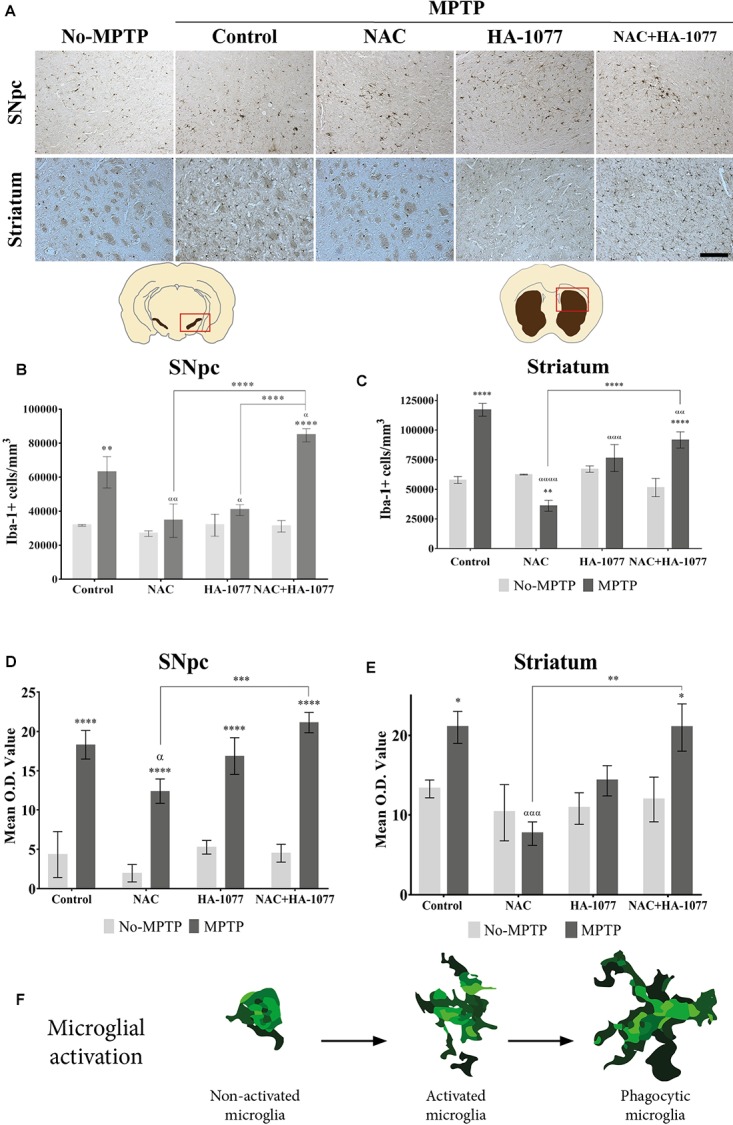
Iba-1+ cell count depends on different treatments in old-Parkinsonian mice. **(A)** Immunohistochemical staining with Iba-1 of coronal sections of SNpc and striatum (Magnification 20×, Scale bar = 100 μm). **(B)** Quantification of Iba-1+ cells in the SNpc by immunostaining analysis. The results showed a significant increase of Iba-1+ cells in MPTP treated mice (^∗∗^*p* = 0.002) and very significant in MPTP+NAC+HA-1077 (^∗∗∗∗^*p* < 0.0001) compared with their control groups. MPTP vs. MPTP+NAC (αα = 0.0020); vs. MPTP+HA-1077 (α = 0.0196); and, vs. MPTP+NAC+HA-1077 (α = 0.0222). MPTP+NAC+HA-1077 vs. MPTP+NAC (^∗∗∗∗^*p* < 0.0001) and vs. MPTP+HA-1077 (^∗∗∗∗^*p* < 0.0001). **(C)** Mean O.D. value measurement for Iba-1+ cells in the SNpc for the morphological changes analysis. A very significant increase was found in MPTP, MPTP+NAC, MPTP+HA-1077 and MPTP+NAC+HA-1077 (^∗∗∗∗^*p* < 0.0001) compared to their respective No-MPTP groups. MPTP vs. MPTP+NAC (α = 0.0344) and MPTP+NAC vs. MPTP+NAC+HA-1077 (^∗∗∗^*p* = 0.0004). **(D)** Iba-1+ cells quantification in the striatum. It was observed a significant increase of Iba-1+ cells in MPTP (^∗∗∗∗^ *p* < 0.0001) and MPTP+NAC+HA-1077 (^∗∗∗∗^*p <* 0.0001). In contrast, a significant decrease in the number of Iba-1+ cells was observed in the MPTP+NAC group compared to its control group (^∗∗^*p* = 0.0011). MPTP vs. MPTP+NAC (α < 0.001); vs. MPTP+HA-1077 (α = 0.0001); vs. MPTP+NAC+HA-1077 (α < 0.0001). **(E)** Mean O.D. value measurement for Iba-1+ cells in the striatum. MPTP (^∗^*p* = 0.0215) and MPTP+NAC+HA-1077 (^∗^*p* = 0.0150) showed a significant increase in the Mean O.D. value compared to No-MPTP groups (Control and HA-1077, respectively). MPTP vs. MPTP+NAC (ααα = 0.0005) and MPTP+NAC vs. MPTP+NAC+HA-1077 (^∗∗^*p* = 0.0011). **(F)** Illustration of microglial activation profiles found in the different experimental groups: non-activated microglia, activated microglia and phagocytic microglia.

In order to evaluate the anti-inflammatory properties of the combined and individual treatments in microglial activation, an immunohistochemical staining was performed in the ventral midbrain and in the striatum for Iba-1 (Figure 3A). Microglial activation was statistically very significant in the SNpc of MPTP (*p* = 0.002) and MPTP+NAC+HA-1077 (*p* < 0.0001), compared to their respective No-MPTP groups (Figure 3B). The morphological analysis by measuring the Mean O.D. value of Iba-1+ cells in the SNpc revealed that all Parkinsonian treated mice showed a very significant increase compared to their respective No-MPTP groups (*p* < 0.0001, Figure 3C and Supplementary Figures S4i–viii).

In the striatum (Figure 3D), there was also a statistically significant increase in Iba-1+ cells in the MPTP (*p* < 0.0001) and MPTP+NAC+HA-1077 (*p* < 0.0001) groups compared to their respective control groups. The number of Iba-1+ cells of MPTP+NAC and MPTP+HA-1077 groups in the striatum was not significantly increased compared to their control groups. These results support those obtained in relation to dopaminergic neuronal death and highlight the anti-inflammatory effect of individual treatment with NAC or with HA-1077. In contrast, Iba-1+ cells in the MPTP+NAC group was significantly lower compared to the NAC No-MPTP group (*p* = 0.0011).

Morphological analysis in the striatum, showed an significant increase in O.D. value in MPTP (*p* = 0.0215) and MPTP+NAC+HA-1077 (*p* = 0.0150) groups compared to their control animals (Figure 3E and Supplementary Figures S4ix–xvi). Interestingly, common morphological patterns were observed in the different stages of microglial activation after the MPTP insult: (i) non-active microglia; (ii) active microglia, and (iii) phagocytic microglia depending on the treatment (Figure 3F).

### The Combined Treatment With NAC+HA-1077 Increases Reactive Astrogliosis in the Striatum

**FIGURE 4:**
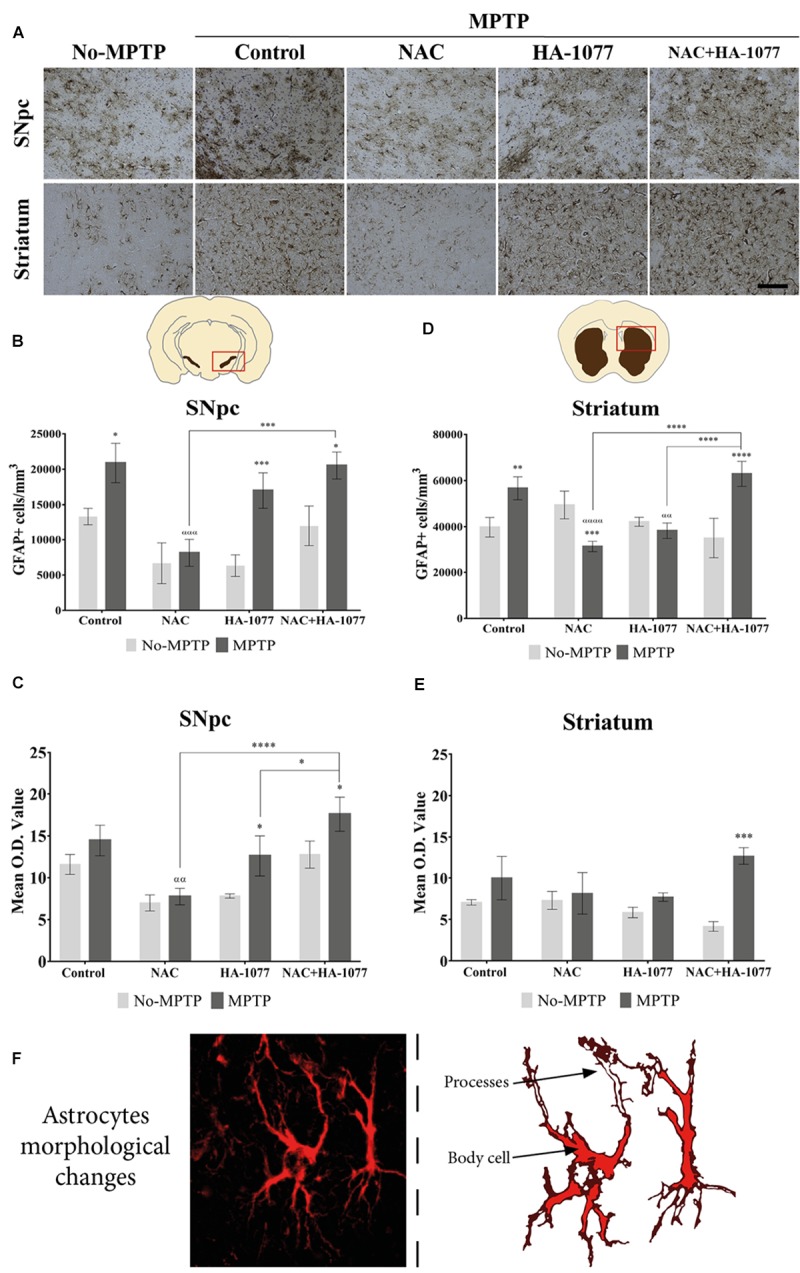
GFAP expression in the SNpc and striatum in brains of old MPTP-intoxicated mice under different treatments. **(A)** Representative photomicrographs of GFAP immunostaining in the SNpc and striatum (Magnification 20×, Scale bar = 100 μm). **(B)** Quantification of GFAP+ cells/mm^3^ in the SNpc. Significant differences were observed in MPTP (^∗^*P* = 0.0235), MPTP+HA-1077 (^∗∗∗^*p* = 0.0009) and MPTP+NAC+HA-1077 (^∗^*p* = 0.0217). MPTP vs. MPTP+NAC (α = 0.0001) and MPTP+NAC vs. MPTP+NAC+HA-1077 (ααα = 0.0002). **(C)** Mean O.D. value measurement for GFAP+ cells in the SNpc for astrocytes’ morphological changes analysis. Significant differences were found in MPTP+HA-1077 (*p* = 0.0208) and MPTP+NAC+HA-1077 (*p* = 0.0249) compared with their No-MPTP groups. MPTP vs. MPTP+NAC (αα = 0.0043). MPTP+NAC+HA-1077 vs. MPTP+NAC (^∗∗∗∗^*p* < 0.0001); and, vs. MPTP+HA-1077 (^∗^*p* = 0.0196). **(D)** Analysis of GFAP positive cells number/mm^3^ in the striatum. GFAP+ cells density was significantly increased in MPTP (^∗∗^*p* = 0.0023) and MPTP+NAC+HA-1077 (^∗∗∗∗^*p* < 0.0001) groups compared with their controls, while a significant decrease was observed in MPTP+NAC group (^∗∗∗^*p* = 0.0005) compared with its control group. MPTP vs. MPTP+NAC (αααα < 0.0001) and, vs. MPTP+HA-1077 (αα = 0.0035). MPTP+NAC+HA-1077 vs. MPTP+NAC (^∗∗∗∗^*p* < 0.0001) and, vs. MPTP+HA-1077 (^∗∗∗∗^*p* < 0.0001). **(E)** Mean O.D. value measurement for GFAP+ cells in the striatum for astrocytes’ morphological changes analysis. MPTP+NAC+HA-1077 (^∗^*p* = 0.0150) showed a significant increase in the Mean O.D. value compared to No-MPTP groups (Control and HA-1077, respectively). **(F)** Illustration of active astrocyte found in the different experimental groups characterized by ramified processes and body cell hypertrophy.

In order to analyze the effect of the different treatment on astrocytes’ activation, immunohistochemical staining for GFAP was performed (Figure 4A). At the SNpc level (Figure 4B), significant differences were found in GFAP expression in the MPTP (*p* = 0.0235), MPTP+HA-1077 (*p* = 0.0009), and MPTP+NAC+HA-1077 (*p* = 0.0217) groups compared with their No-MPTP groups. The astrocytes’ morphological analysis in the SNpc showed a slight increase of Mean O.D. value of the GFAP immunostaining in the MPTP, MPTP+HA-1077 and MPTP+NAC+HA-1077 groups compared to their respective No-MPTP groups in the SNpc (Figure 4C and Supplementary Figures S5i–viii).

In addition, striatal GFAP expression was significantly increased in MPTP (*p* = 0.0023) and very significantly increased in MPTP+NAC+HA-1077 (*p* < 0.0001) compared with their respective control groups (Figure 4D). Importantly, NAC treatment in old-Parkinsonian mice produced a very significant decrease in the number of GFAP+ cells/mm^3^ in the striatum (*p* = 0.0005). The study of morphological changes of astrocytes in the SNpc showed significant changes in MPTP+HA-1077 (*p* = 0.0208) and MPTP+NAC+HA-1077 (*p* = 0.0249) compared with their No-MPTP groups. Moreover, in the striatum showed that mean O.D. value was significantly increased in MPTP+NAC+HA-1077 (*p* = 0.0002) group compared to its No-MPTP group (Figure 4E and Supplementary Figures S5ix–vi). Activated astrocytes presented numerous and ramified processes as well as cell body hypertrophied depending on the treatment group (Figure 4F).

### Astroglial Activation Processes Involved in the Dopaminergic Neuronal Death

**FIGURE 5:**
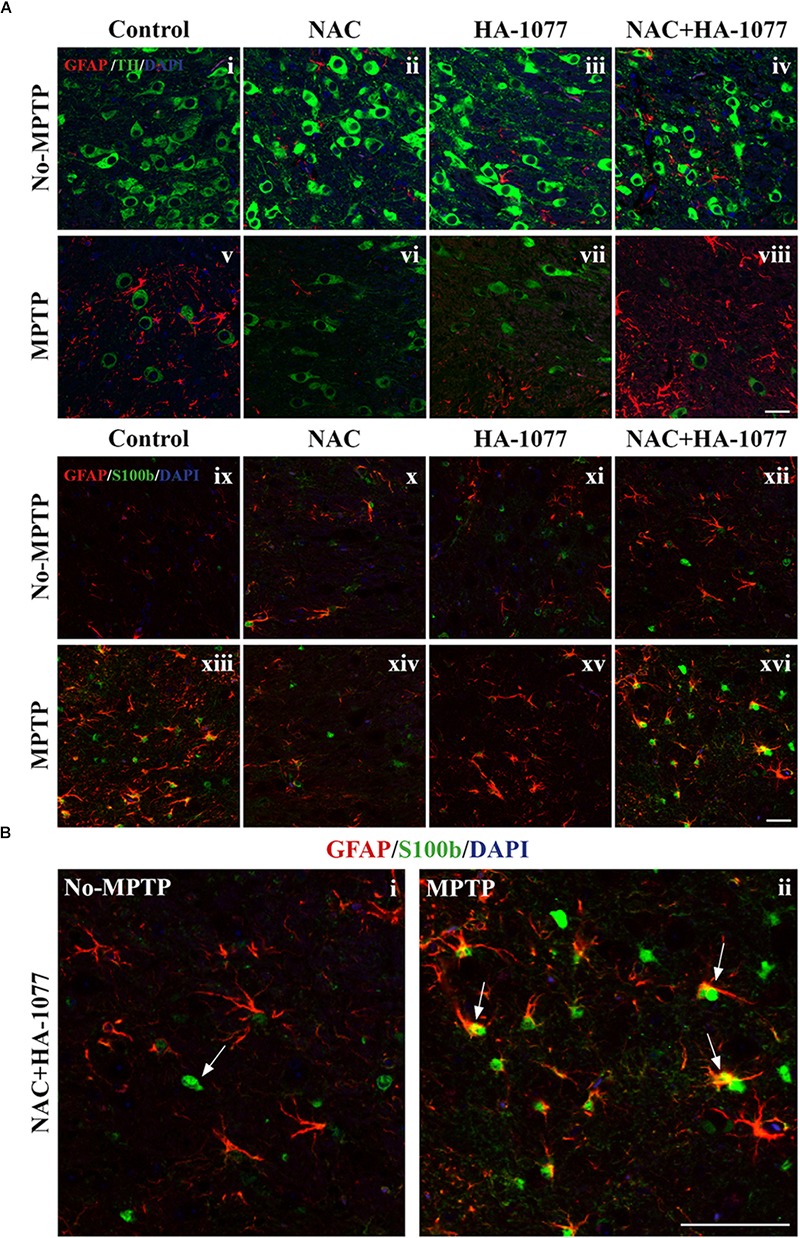
Study of GFAP and S100b with TH in SNpc. **(A)** Representative micrographs of the double immunofluorescence assay for GFAP+TH **(i–viii)** and GFAP+S100b **(ix–xvi)** in the SNpc of the different experimental groups. **(B)** Representative figures for GFAP and S100b in the MPTP+NAC+HA-1077 group in which different astrocyte profiles were observed: S100b expressed in the nucleus and S100b co-located with GFAP in perinuclear cytoplasm. (Magnification 63×, Scale bar = 50 μm).

To get deeper into the analysis of astroglial response among treatments, a double immunofluorescence was performed for TH and GFAP markers at the SNpc level. In Figure 5Ai–viii, it can be observed an indirect relationship in the expression of these markers in MPTP, MPTP+HA-1077, and MPTP+NAC+HA-1077 groups. Thus, a decrease in the expression of TH + cells is accompanied by an increase in the expression of GFAP+ cells. On the other hand, in the MPTP+NAC group, this relationship was not as pronounced.

In addition to the increase in GFAP expression, it has been shown that S100b levels increase during reactive astrogliosis in SNpc and striatum of PD patients, suggesting that this protein could play an important role in disease progression (Jackson-Lewis and Przedborski, 2007). Thus, immunofluorescence labeling for astrocyte markers GFAP and S100b was performed in the striatum. The double immunolabeling of these two markers revealed the existence of different profiles of astrocytes depending on the protein subcellular localization (Figure 5Aix–xvi). One astrocyte’s profile is characterized by the presence of S100b only in the nucleus which is predominantly found in No-MPTP, MPTP+NAC, and MPTP+HA-1077. In MPTP and MPTP+NAC+HA-1077, the expression of S100b was found in the nucleus and in the perinuclear cytoplasm co-localizing with GFAP (Figure 5B). Taking all this data together, GFAP+S100b co-localization could have an important role in the dopaminergic neuronal death after MPTP intoxication in old mice.

### Indirect Mediation of the JNK Pathway in Astroglial Response

Previous studies have demonstrated that dopaminergic neuronal death in the MPTP model is principally regulated by the JNK signaling pathway activation. It has been reported an increase in the p-JNK levels after MPTP intoxication mainly in response to the associated oxidative stress processes (Saporito et al., 1999; Lotharius et al., 2005; Pan et al., 2009). We have demonstrated that NAC treatment, an inhibitor of JNK, has a neuroprotective effect in old-Parkinsonian mice. Thus, we wanted to investigate whether the neuronal cell death was promoted by JNK signaling pathway activation in the SNpc.

**FIGURE 6:**
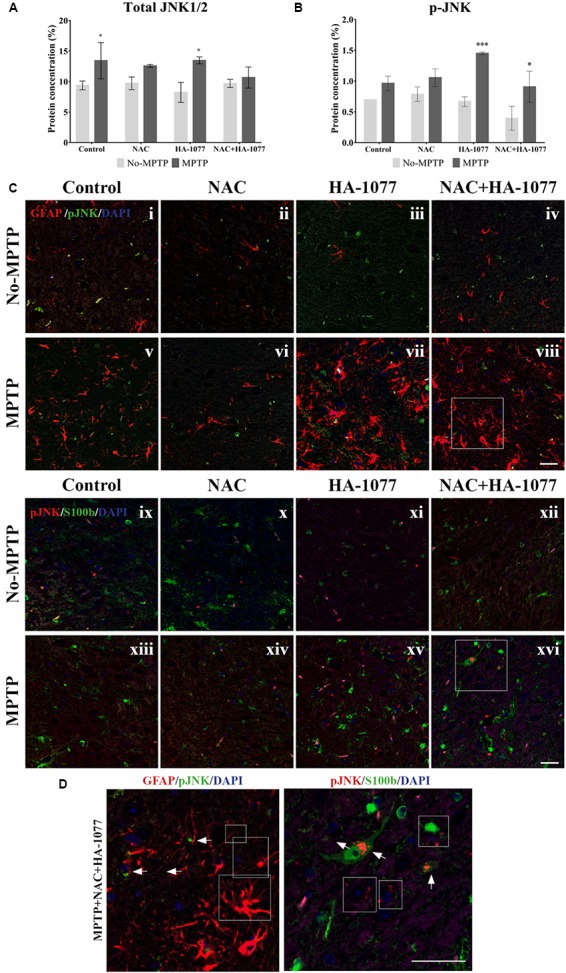
Quantification of total and phospho-JNK1/2 expression by ELISA in midbrains of old-Parkinsonian mice. **(A)** There was a significant increase in protein concentration (%) of total JNK1/2 in MPTP (^∗^*p* = 0.0321) and MPTP+HA-1077 (^∗^*p* = 0.0129) groups compare with their controls. **(B)** Quantification of p-JNK concentration (%) by ELISA in midbrains of old-Parkinsonian mice. p-JNK expression was significantly increase in MPTP+HA-1077 (^∗∗∗^*p* = 0.0007) and MPTP+NAC+HA-1077 (^∗^*p* = 0.0219). **(C)** Confocal images for GFAP and p-JNK in SNpc **(i–viii)**. GFAP immune-reactive cells (red) were significantly increased in the MPTP **(v)**, MPTP+HA-1077 **(vii)** and MPTP+NAC+HA-1077 **(viii)** treated mice compared to their control groups (**i,iii,iv**, respectively). Phosphorilated JNK (pJNK, red) was not found in the GFAP+ cells’ nucleus. Instead, this apoptotic protein appeared in the perinuclear region of the SNpc neurons. **(D)** Representative confocal images for S100b and p-JNK in SNpc **(ix–xvi**). (Magnification 63×, Scale bar = 50 μm).

Then, quantification of total JNK and p-JNK levels was performed by ELISA assay in the midbrain extracts (Figures 6A,B). The results showed a significant increase in total JNK levels in the MPTP group (*p* = 0.0321) and MPTP group treated with HA-1077 (*p* = 0.0129) compared to their respective controls. Furthermore, a significant increase in p-JNK levels was observed in the MPTP+HA-1077 group (*p* = 0.0007) and, reinforcing previous results, in the MPTP+NAC+HA-1077 group (*p* = 0.0219).

In addition, immunofluorescence staining for GFAP and S100b with p-JNK was performed at the SNpc level (Figure 6C) in order to examine possible changes in MAPK signaling pathway in astrocytes. In Figures 6i–viii, all MPTP treated groups, except those treated with NAC, showed hypertrophic astrocytes with an increase of GFAP, which does not co-localize with p-JNK. In Figures 6Cix–xvi, S100b expression was increased in the SNpc of MPTP, MPTP+HA-1077 and MPTP+NAC+HA-1077 groups, which co-localized with p-JNK (for more details see Supplementary Figure S6) (Figure 6D). These results suggest that the JNK pathway does not directly mediate astroglial activation. A deeper analysis of p-JNK expression in neurons may help in understanding its potential role in astroglial activation.

## Discussion

Recent studies in the literature have reported that inflammatory processes mediated by microglia and astrocytes might be an important key in the development and progression of PD (Wang et al., 2015; Tiwari and Pal, 2017). These mechanisms are exacerbated during aging, where there are a cell degeneration and glial dysfunction (Collier et al., 2017). Taking this background as a starting point, in the present study we decided to examine the possible synergistic effect of the combined treatment composed by an anti-inflammatory (HA-1077) and an antioxidant (NAC). Both HA-1077 and NAC, have been individually described to have beneficial effects on dopaminergic neuronal death in young MPTP intoxicated mice (Pan et al., 2009; Barcia et al., 2012). In *post-mortem* studies, we first analyzed dopaminergic neuronal death by TH immunostaining in the SNpc and the striatum (Figure 2). At the SNpc level, a significant decrease in the number of dopaminergic neurons in old MPTP treated mice was found (Figures 2B,D). On the other hand, in the striatum, a very significant decrease was observed in TH expression in DA fibers in MPTP, MPTP+HA-1077, and MPTP+HA-1077+NAC groups, but not in MPTP+NAC group (Figures 2C,E). These data may be explained by the fact that neurodegeneration, induced by MPTP injections, first affects the dopaminergic terminals in the striatum which project from the neuronal body at SNpc level (Morales et al., 2016).

These results are impressive because it was not expected to have a significant neuronal death within the combined treatment. These unexpected results were supported by the study of microglial activation. In the SNpc and in the striatum, an increase in the number of Iba-1+ cells was shown in all MPTP groups, with the exception of MPTP+NAC group (Figures 3B,D). In addition, morphological changes related with the different states of microglia cells under different pathological conditions were identified by measurement of Mean O.D value (Figures 3C,E). In No-MPTP groups, microglia was mainly not activated; in MPTP and MPTP+HA-1077 groups activated microglia was observed whereas phagocytic microglia was only identified in MPTP+NAC+HA-1077 group (Figure 3 and Supplementary Figure S4). Barcia and co-workers also described this relationship between the exacerbation of neuronal death and microglial activation (Barcia et al., 2012).

On the other hand, the quantification of GFAP+ cells resulted significantly increased in MPTP+HA-1077 and MPTP+NAC+HA-1077 in the SNpc (Figure 4B). In the striatum, significant differences were observed in the groups treated with MPTP and MPTP+NAC+HA-1077 (Figure 4D). These data are consistent with the description of inflammatory events following damage in the CNS. The vicious cycle begins with the release of pro-inflammatory cytokines by dopaminergic neurons that stimulate microglia from an anti-inflammatory state to a pro-inflammatory state. If the damage persists, an uncontrolled release of pro-inflammatory cytokines is produced which stimulates astrocytes to a state of reactive astrogliosis and, consequently, exacerbating neuronal death.

The pro-neurotoxin MPTP is converted to the neurotoxin MPP+ by astrocytes and then released to dopaminergic neurons (Huang et al., 2017). In order to evaluate if the combined treatment was exacerbating reactive astrogliosis (and, in consequence, dopaminergic neuronal death) immunofluorescence staining for GFAP with TH and S100b in the SNpc was performed. In concordance with other studies, we found an increase of GFAP expression whereas there was a decrease in the number of TH+ neurons in MPTP, MPTP+HA-1077, and MPTP+NAC+HA-1077 groups (Huang et al., 2017).

Moreover, results showed astrocytes with different expression profiles depending on treatment: both the MPTP and the MPTP+NAC+HA-1077 had an increase in cytoplasmic S100b expression, whereas, in No-MPTP and MPTP+NAC groups, S100b was found only in the nucleus (Figure 5).

The following steps in our study were focused on answering why does a combination of drugs, which individually have neuroprotective effects, produce a toxic effect in old-Parkinsonian mice. One of the main basis of our hypothesis is supported by the fact that the elderly brains are more vulnerable to intoxication because, among other factors, they have a basal inflammatory condition that makes them more susceptible to cell degeneration (Savva et al., 2009). All our data discussed pointed out that NAC had a beneficial effect; HA-1077 had a toxic one and the combination of both showed a very toxic effect on neuronal death and glial associated response. Based on these results, we further study the possible metabolic pathway that could be affected by the combined treatment.

Many studies suggest that one of the possible metabolic pathways involved during a cellular stress condition (in this case by the administration of a neurotoxin) may be MAPKinases pathway (Kim and Choi, 2015). MAPKinases are a protein family composed by ERK, p38, and JNK. This last protein caught our attention for being the target of one of our drugs, NAC (Pan et al., 2009). Then, we decided to analyze JNK expression in the different experimental groups and, surprisingly, an increase of its phosphorylated state was observed in thegroup MPTP+HA-077 and MPTP+HA-1077+NAC, but not in the group treated with MPTP+NAC (Figures 6A,B).Double immunofluorescence staining in the SNpc level for p-JNK together with GFAP and S100b was performed (Figure 6C). It was observed that p-JNK not co-localized neither with GFAP nor with S100b. These findings suggest that astroglial response could be indirectly mediated by the phosphorylation of JNK in damaged neurons. New research lines could further study the relationship between JNK pathway and neuroinflammation processes after an MPTP injection and, in this way, identified its crucial role in the degeneration of the nigrostriatal dopamine system.

According to the results obtained in the present study, we postulate that the combination of HA-1077 and NAC administrated in the most vulnerable brains, because of aging, has turned out to increase neuroinflammatory processes. With this, we conclude that personalized therapy should be supported in patients with different treatments based on the described data. Therefore, this work opens the door to consider the study of the effects of the combination of common drugs on neuronal death and inflammation, in order to design therapeutic strategies more adapted to PD patients (mainly in elderly people).

## Author Contributions

ALG-M and LC carried out all the experiments and wrote the manuscript. CS-R, CE, and EF-V participated in the experiments. MTH conceived the idea and design of the study, discussed the results, and worked in the manuscript preparation. All authors read and approved the final manuscript.

## Conflict of Interest Statement

The authors declare that the research was conducted in the absence of any commercial or financial relationships that could be construed as a potential conflict of interest.

## Abbreviations

CNS: Central Nervous System
DA: dopaminergic
ERK: Extracellular signal-regulated kinase
GFAP: glial fibrillary acidic protein
Iba-1: ionized calcium-binding adapter molecule 1
JNK: c-Jun NH2-terminal kinase
MAPKs: Mitogen-activated protein kinases
MPTP: 1-methyl-4-phenyl-1,2,3,6-tetrahydropyridine
NAC: N-acetylcysteine
PD: Parkinson’s Disease
ROS: Reactive Oxygen Species
SNpc: Substantia Nigra *pars compacta*
TH: Tyrosine hydroxylase
VTA: Ventral Tegmental Area
