# Supplementary material for: Unexpected Exacerbation of Neuroinflammatory Response After a Combined Therapy in Old Parkinsonian Mice
Source: Front Cell Neurosci. 2018 Nov 30;12:451. doi: 10.3389/fncel.2018.00451 (PMC6284242; doi:10.3389/fncel.2018.00451)
Supplement: Supplementary file 1 [file Table_1.DOCX]

**Supporting information**

Unexpected exacerbation of neuroinflammatory response after a combined therapy in old Parkinsonian mice

Gil-Martínez, Ana-Luisa; Cuenca, Lorena; Sánchez, Consuelo; Estrada, Cristina; Fernández-Villalba, Emiliano and Herrero, María-Trinidad

**Supporting Information Inventory**

| Figure S1, related to Experimental Procedures | page 2 |
| --- | --- |
| Figure S2, related to Experimental Procedures | page 4 |
| Figure S3, related to Results | page 6 |
| Figure S4, related to Figure 2 | page 7 |
| Figure S5, related to Figure 3 | page 8 |
| Figure S6, related to Figure 5 | page 9 |
| Supporting figures legends | page 10 |

**Figure S1.1**


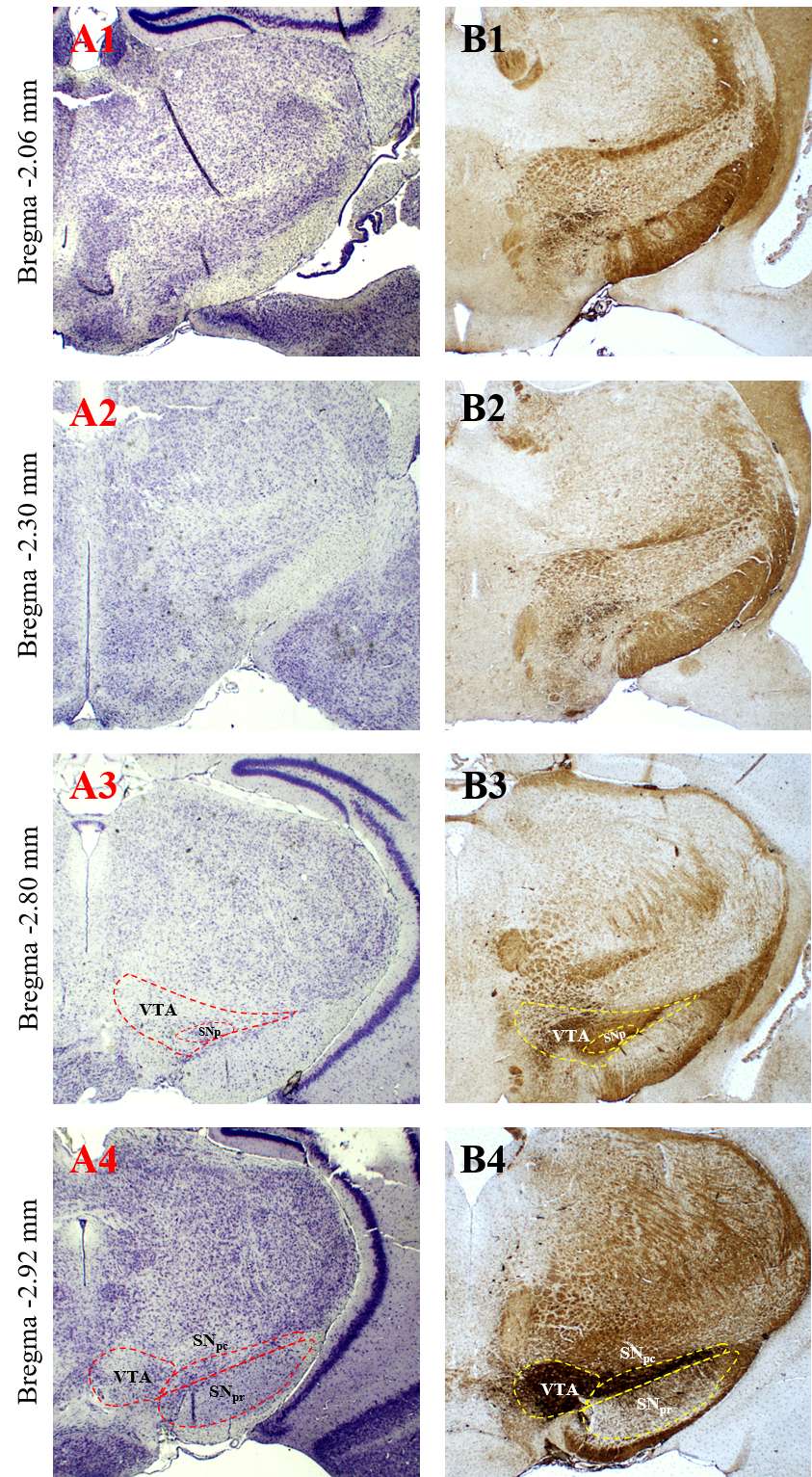


**Figure S1.2**


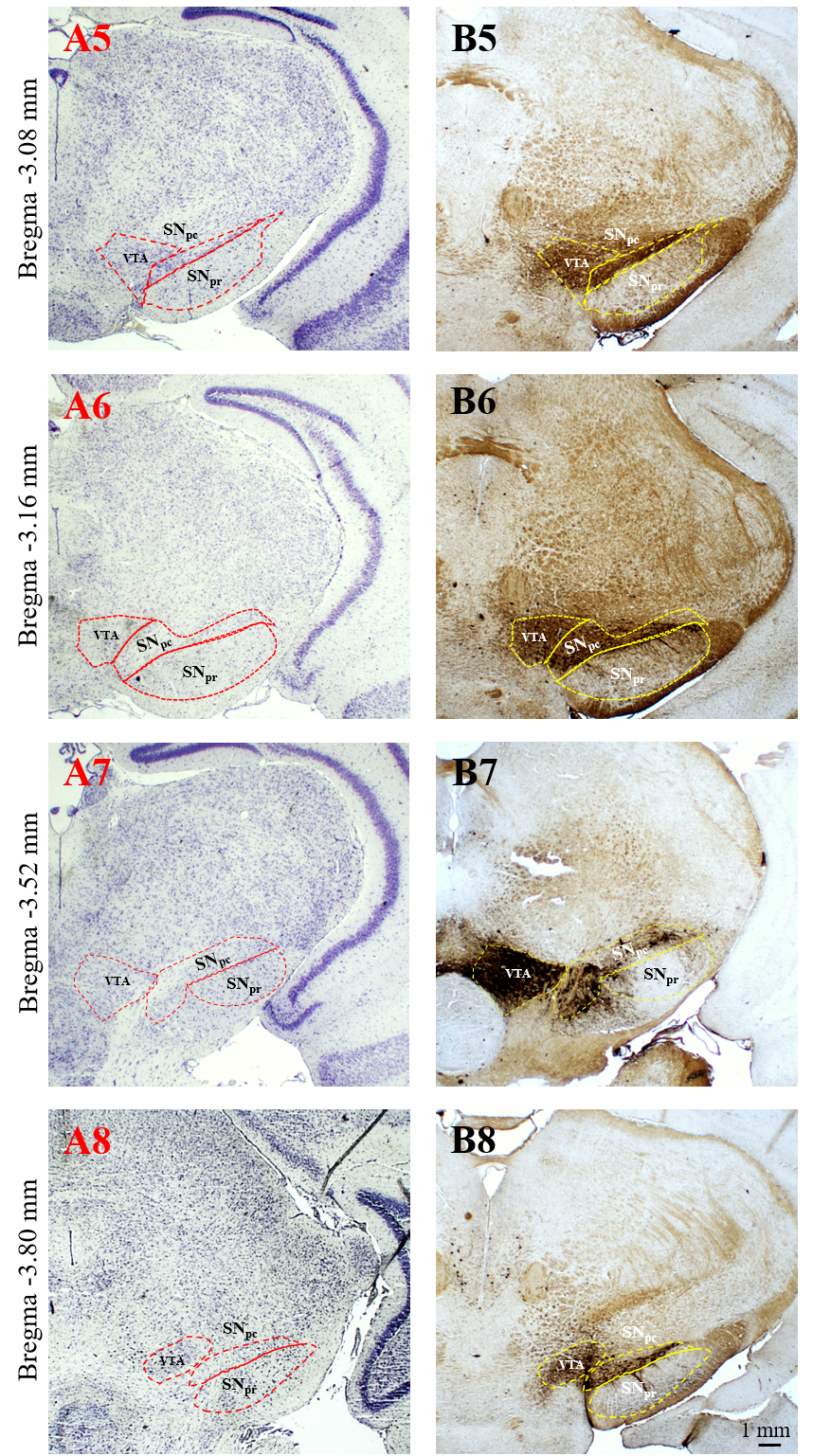


***
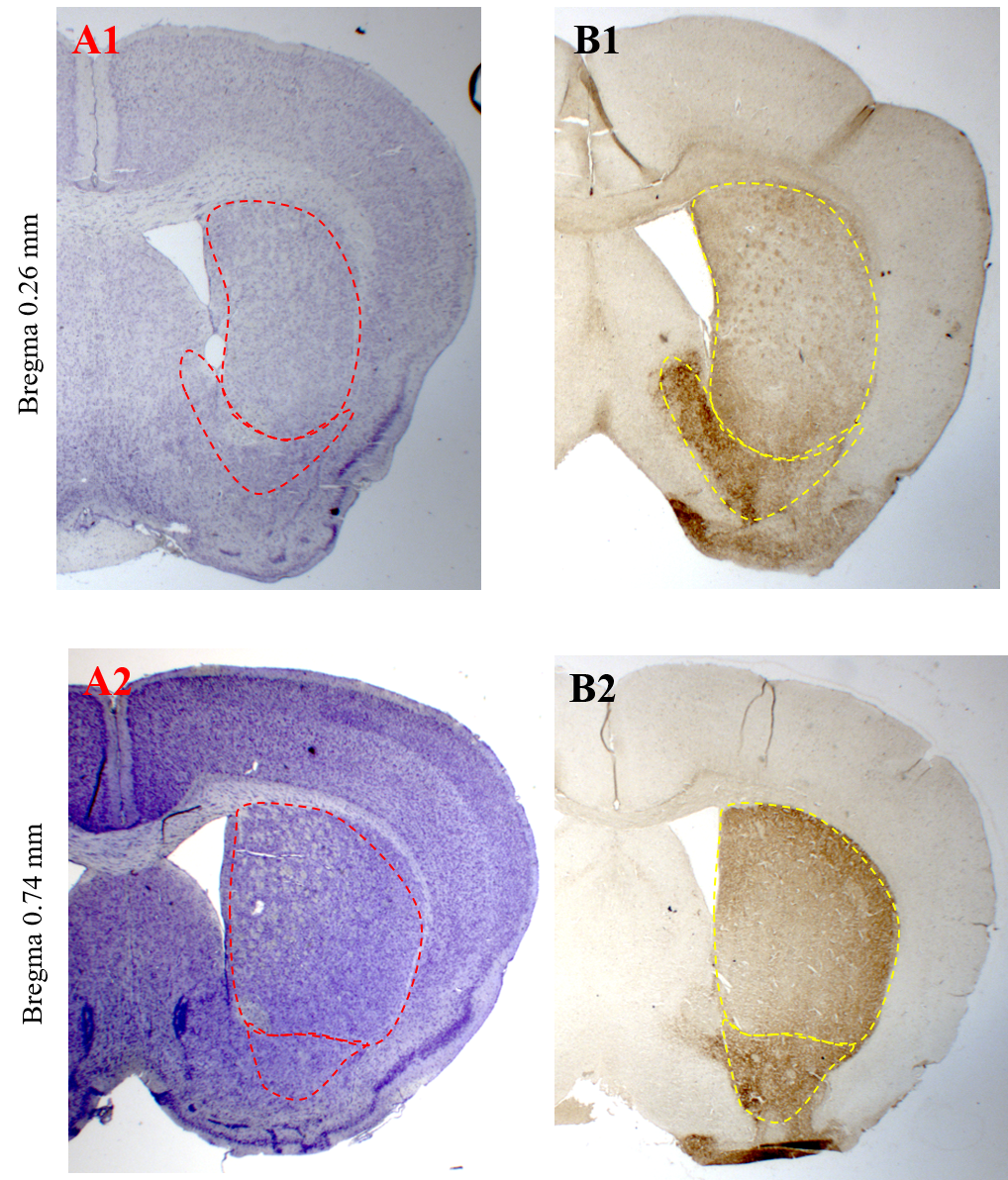
*Figure S2.1**

**Figure S2.2**


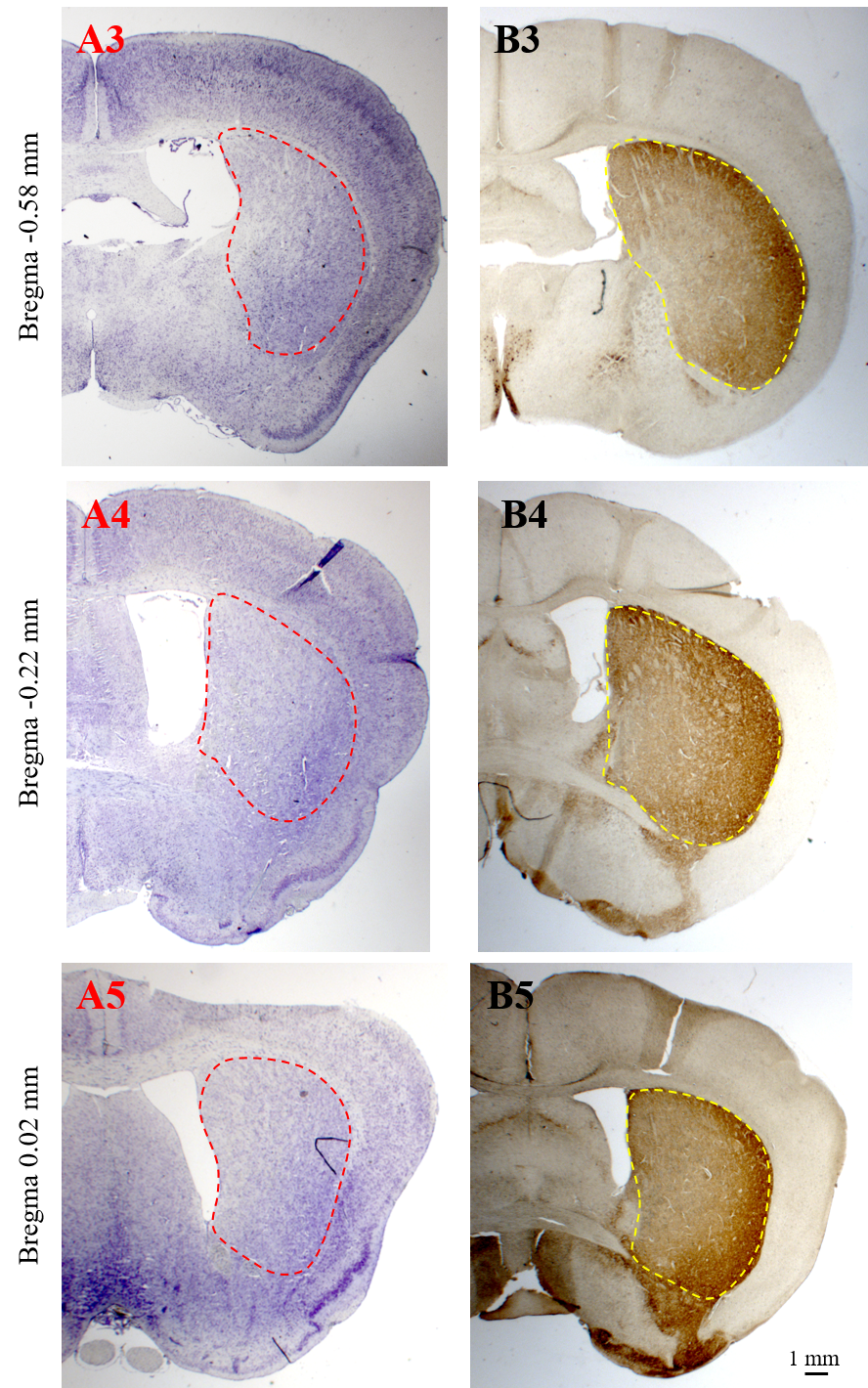


**Figure S3**


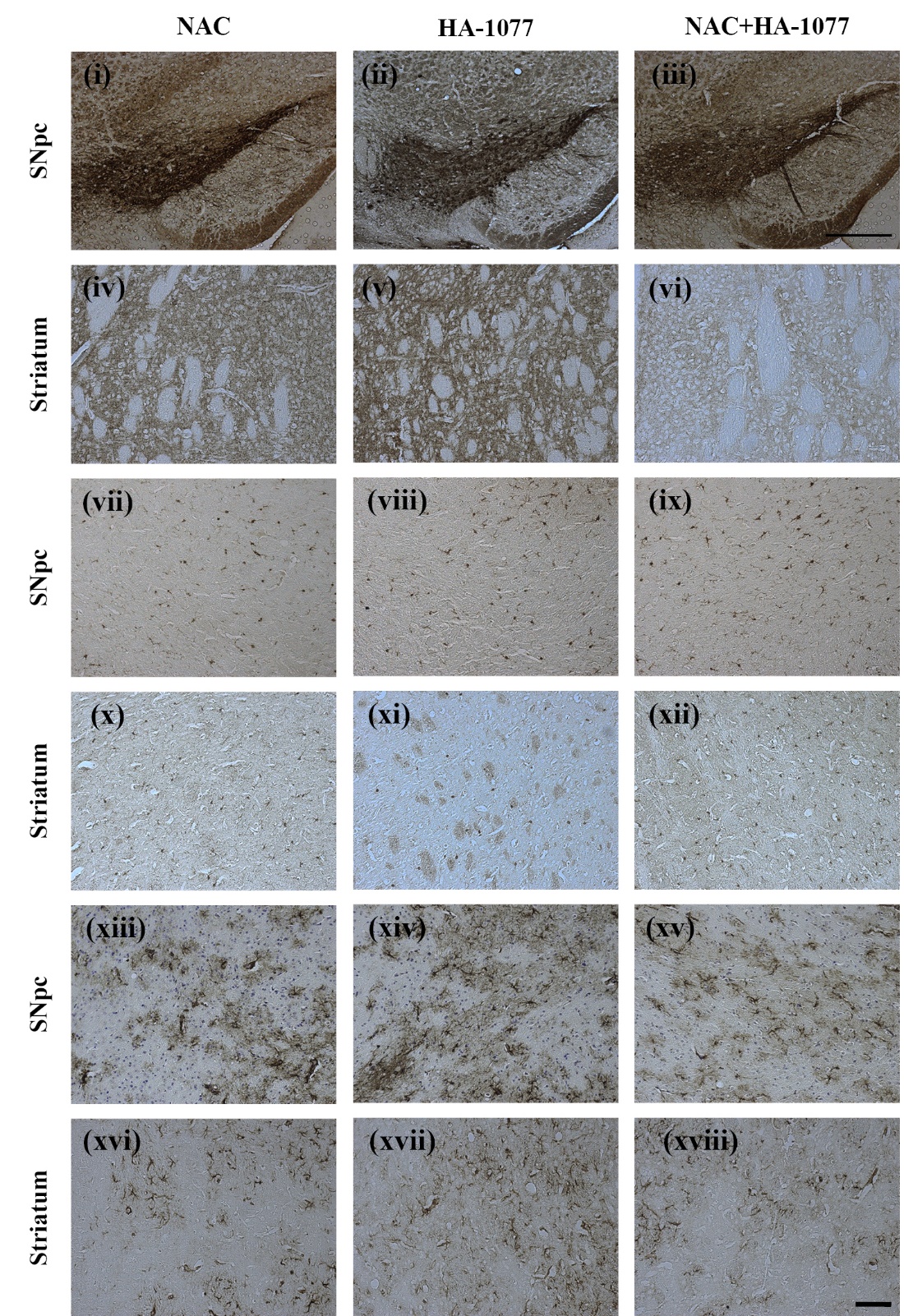


**Figure S4**


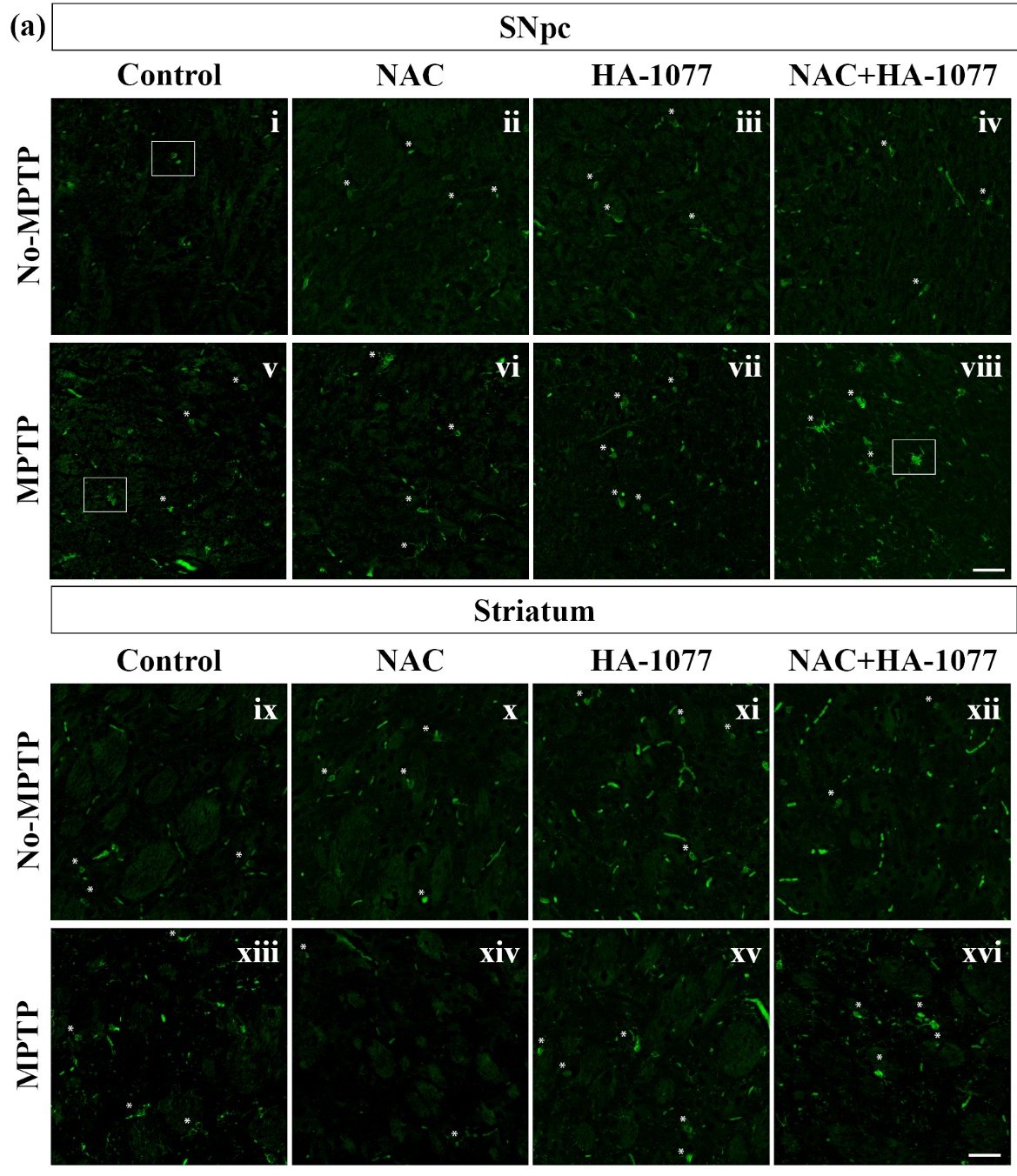


**Figure S5**


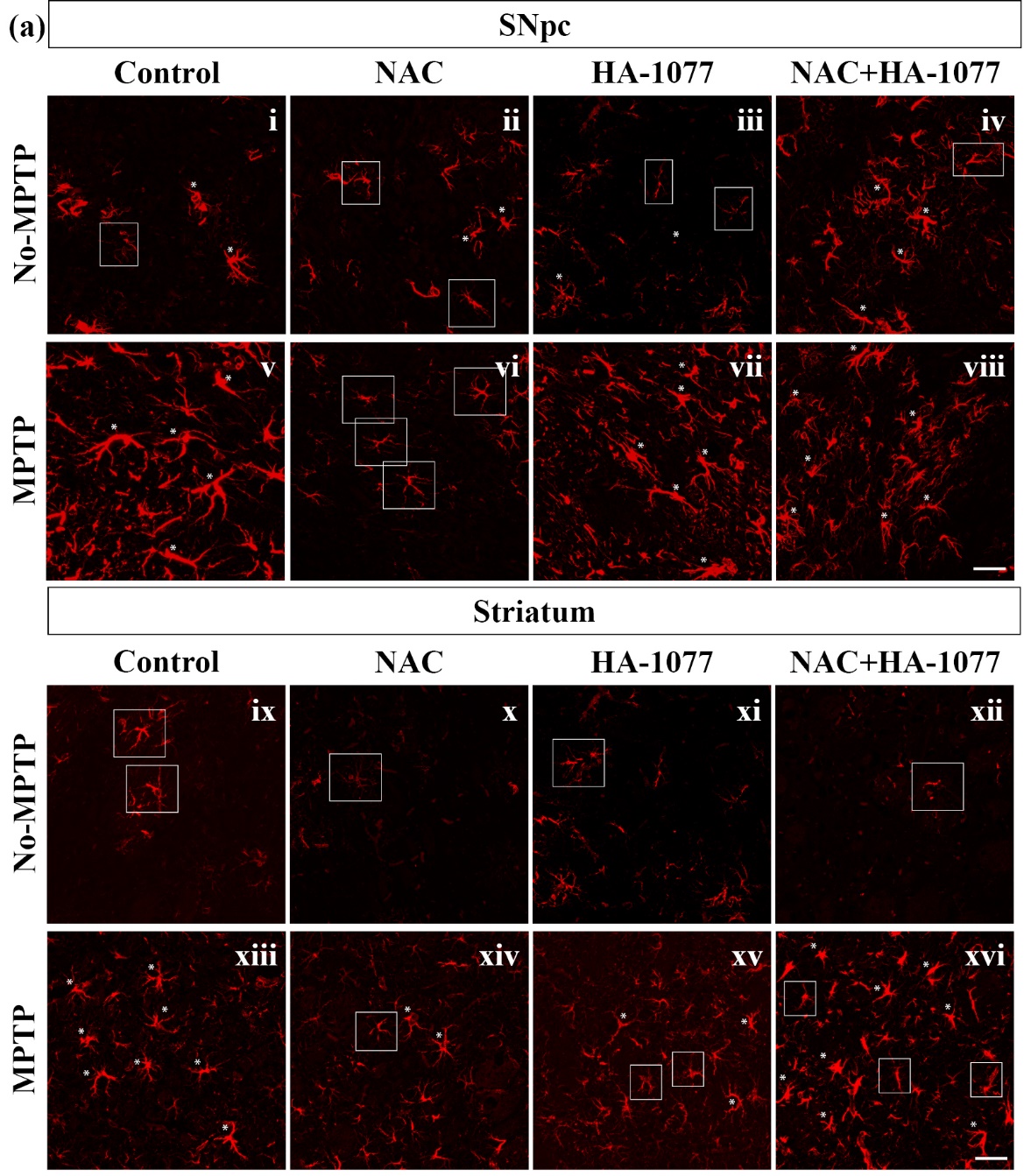


**Figure S6**


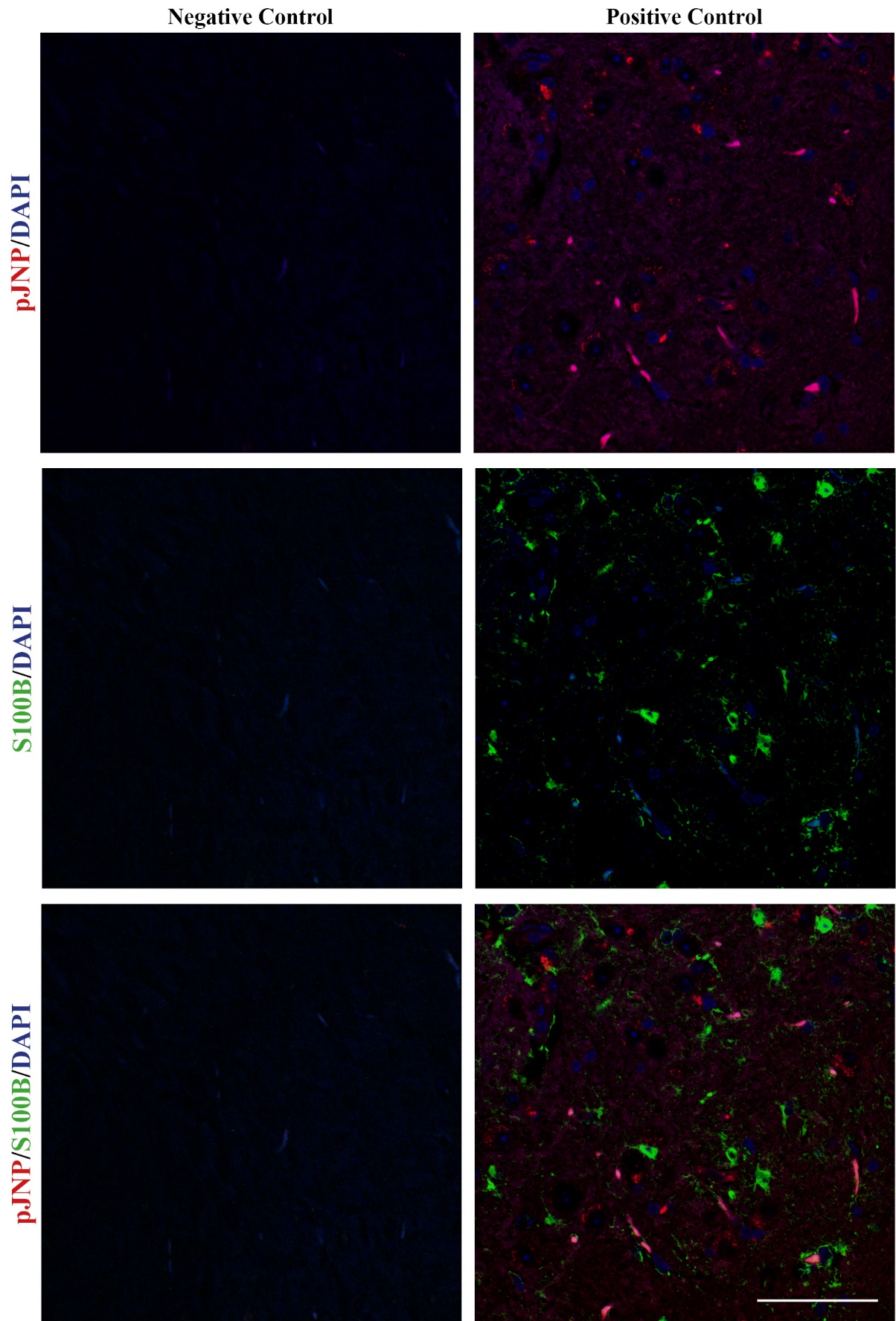


**Supporting figure legends**

**Supplementary Figure 1**

For unbiased quantification of DAB staining, eight serial sections (B1-B8, from rostral to caudal) were chosen as representative slices. SNpc area was located trough the different sections regarding distinguishable anatomical zones surrounding it (VTA and SNpr). Sections entitled A1-A8 were stained for Nissl and sections entitled were TH-immunostained. Scale bar: 1 mm.

**Supplementary Figure 2**

Landmark for striatum were stablished using Nissl stainning representative sections organized from rostral to caudal (A1-A5), on the left side. B series (B1-B5) were immunostained for TH. Scale bar: 1 mm.

**Supplementary Figure 3**

Representative micrographs of No-MPTP groups (NAC; HA-1077 and NAC+HA-1077) labeled with TH (i-iii and iv-vi), Iba-1 (vii-ix and x-xii) and GFAP (xiii-xv and xi-xiii) in the SNpc and striatum, respectively. (i-ii) Magnification 10x, Scale bar = 100 µm. (iv-xiii) Magnification 20x, Scale bar = 100 µm.

**Supplementary Figure 4**

(a) Representative micrographs of Iba-1+ cells in the SNpc (i-viii) and (b) striatum (ix-xvi) (Magnification 63x, Scale bar = 50 µm). White asterisk (*) denote Iba-1+ cells and white frame shows representative cells with described morphological feature.

**Supplementary Figure 5**

FIGURE 2. (a) Representative micrographs of GFAP+ cells in the SNpc (i-viii) and (b) striatum (ix-xvi) (Magnification 63x, Scale bar = 50 µm). White asterisk (*) denote Iba-1+ cells and white frame shows representative cells with described morphological features.

**Supplementary Figure 6**

Positive and negative controls of immunolabeling for S100B and p-JNK.
